# Use of mouthwashes in the management of COVID-19 patients in intensive care units: recommendations and current evidence

**DOI:** 10.31744/einstein_journal/2021CE6419

**Published:** 2021-03-18

**Authors:** Marcos Roberto Tovani-Palone, Erfan Shamsoddin

**Affiliations:** 1 Universidade de São Paulo Faculdade de Medicina de Ribeirão Preto Ribeirão PretoSP Brazil Faculdade de Medicina de Ribeirão Preto, Universidade de São Paulo, Ribeirão Preto, SP, Brazil.; 2 National Institute for Medical Research Development Tehran Iran National Institute for Medical Research Development, Tehran, Iran.

Dear Editor,

According to recent studies, approximately 33% of patients hospitalized with coronavirus disease 2019 (COVID-19) should require intensive care, and up to 20% of those admitted to hospital may need invasive mechanical ventilation.^(^^1^^)^ Considering the performance of multidisciplinary teams composed of physicians, nurses, dentists, physical therapists, and other health professionals at intensive care units (ICU) for treatment of COVID-19 patients,^(^^2^^-^^4^^)^ and evidence pointing to a decrease in viral load of severe acute respiratory syndrome coronavirus 2 (SARS-CoV-2) in saliva through the use of mouthwashes,^(^^5^^,^^6^^)^ some questions on this subject deserve to be discussed in more detail, focusing on safe approaches for the professional teams working in this context, as well as in relation to appropriate care for these patients.

First, based on the existing evidence, the use of mouthwashes in COVID-19 patients admitted to ICU is currently suggested before performing daily routine procedures with a potential risk of generating aerosols, and/or droplet emission during the care of intubated patients, and droplet release from those without intubation, which should undoubtedly contribute to reduce the risks of virus transmission to the team of health professionals at ICU^(^^5^^)^ (Table 1).

**Table 1:** Mouthwashes used in the treatment of COVID-19 patients admitted to intensive care units

Recommended mouthwashes	Concentration	Main actions
C31G (SAVVY^®^)		Reduction of oral microorganisms in patients with COVID-19
Cetylpyridinium chloride	0.05%	Possible antiviral effect on SARS-CoV-2
Povidone iodine	0.2%,0.4%, or 0.5%	Reduction of the SARS-CoV-2 viral load, contributing to the control of oral hygiene and respiratory tract
Hydrogen peroxide	1.5% or 3%	Reduction of salivary viral load of SARS-CoV-2
Chlorhexidine	0.12%	Suppression of SARS-CoV-2 for 2 hours after use, improvement of symptoms in COVID-19 patients presenting changes in oral flora, and reduction of ventilator-associated pneumonia

Source: adapted from Moosavi MS, Aminishakib P, Ansari M. Antiviral mouthwashes: possible benefit for COVID-19 with evidence-based approach. J Oral Microbiol. 2020;12(1):1794363. Review;^(^^5^^)^ Vergara-Buenaventura A, Castro-Ruiz C. Use of mouthwashes against COVID-19 in dentistry. Br J Oral Maxillofac Surg. 2020;58(8):924-7. Review.^(^^6^^)^

**Figure 1:**
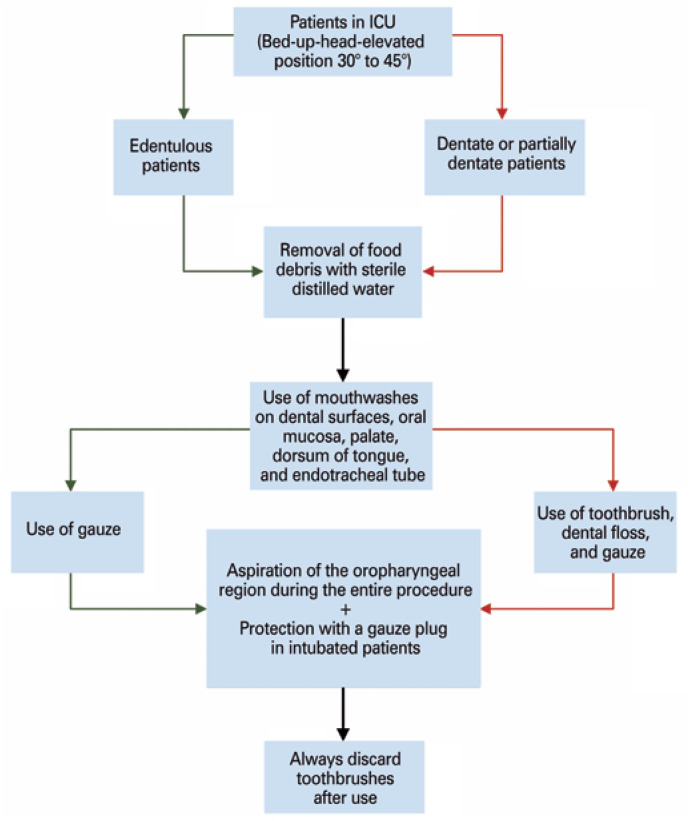
Recommendations for oral hygiene technique in COVID-19 patients admitted to intensive care units. Red arrows: applicable to dentate or partially dentate patients; green arrows: applicable to edentulous patients; black arrows: applicable to both groups

Source: adapted from Agência Nacional de Vigilância Sanitária (ANVISA). Gerência de Vigilância e Monitoramento em Serviços de Saúde. Gerência Geral de Tecnologia em Serviços de Saúde. Medidas de prevenção de infecção relacionada à assistência à saúde. v. 4. Brasília (DF): Ministério da Saúde; 2017. p. 92 [Série Segurança do Paciente e Qualidade em Serviços de Saúde] [cited 2020 Dec 26]. Disponível em: https://www20.anvisa.gov.br/segurancadopaciente/index.php/publicacoes/item/caderno-5;^(^^7^^)^ Conselho Federal de Odontologia (CFO). Recomendações AMIB/CFO para atendimento odontológico COVID-19: Comitê de Odontologia AMIB/CFO de enfrentamento ao COVID-19. Brasília (DF): AMIB/CFO; 2020 [cited 2020 Dec 26]. Disponível em: http://www.crosp.org.br/uploads/arquivo/d45f45bec26af5e60711423292623321.pdf.^(^^8^^)^ ICU: intensive care units.

Another important point concerning the use of mouthwashes in this group of patients is the contribution to improve of systemic problems associated with oral microbial flora, as well as to prevent the occurrence of nosocomial pneumonia associated with mechanical ventilation, leading to a probable reduction in the length of hospital stay^(^^5^^)^ (Figure 1). Also, the possibility of reducing the risk of cross-infection among hospitalized patients during the pandemic is expected as a secondary benefit.^(^^6^^)^

It is worth mentioning that for effective oral hygiene in COVID-19 patients admitted to ICU, some criteria must be carefully met. It is of utmost importance that each health care organization fits within its reality, regarding the combination of mouthwashes chosen. In figure 1, we describe a proposal of steps to carry out this procedure.

Additionally, within this proposal, positioning patients with raised head mainly aims to avoid cases of pneumonia - due to healthcare-associated infections, while improving respiratory parameters.^(^^7^^)^Furthermore, after initial aspiration of secretions above the cuff in intubated patients, the use of a gauze plug is recommended to complement the lung protection, which should be performed before the use of mouthwashes.^(^^8^^)^

However, even with fundamental advances in protocols for the management of ICU COVID-19 patients, the professional teams involved should always prioritize procedures with a lower risk of generating aerosols or droplet emission, which would provide greater security in the hospital environment. Moreover, the performance of intensive care dentists becomes essential to the comprehensive care of these patients.
