# Supplementary material for: Use of mouthwashes in the management of COVID-19 patients in intensive care units: recommendations and current evidence
Source: Einstein (Sao Paulo). 2021 Mar 18;19:eCE6419. doi: 10.31744/einstein_journal/2021CE6419 (PMC7971153; doi:10.31744/einstein_journal/2021CE6419)
Supplement: Translation

# Translation: Uso de colutórios no manejo de pacientes com COVID-19 em unidades de terapia intensiva: recomendações e evidências atuais

**Authors:** Marcos Roberto Tovani-Palone, Erfan Shamsoddin

**Affiliations:** 1 Ribeirão PretoSP Brasil Faculdade de Medicina de Ribeirão Preto, Universidade de São Paulo, Ribeirão Preto, SP, Brasil.; 2 Tehran Iran National Institute for Medical Research Development, Tehran, Iran.

Caro Editor,

Segundo estudos recentes, cerca de 33% dos pacientes internados com a doença pelo coronavírus 2019 (COVID-19) devem requerer atendimento em unidades de terapia intensiva (UTI), além de até 20% do total de internados poder necessitar do uso de ventilação mecânica invasiva.^(^^1^^)^ Tendo em vista a atuação de equipes multiprofissionais compostas por médicos, enfermeiros, dentistas, fisioterapeutas e outros profissionais de saúde em UTI para o tratamento de pacientes com COVID-19^(^^2^^-^^4^^)^ e a existência de evidências que apontam para uma diminuição da carga viral do coronavírus da síndrome respiratória aguda grave 2 (SARS-CoV-2) na saliva mediante o uso de colutórios,^(^^5^^,^^6^^)^ alguns pontos inerentes a esse assunto merecem ser discutidos em mais detalhes, com foco em abordagens seguras para as equipes profissionais atuantes nesse contexto, bem como em relação à assistência adequada a esses pacientes.

Primeiro, com base nas evidências existentes, tem-se que, atualmente, sugere-se o uso de colutórios em pacientes com COVID-19 internados em UTI previamente à realização de procedimentos diários de rotina com risco potencial de gerar aerossóis e/ou emissão de gotículas durante a assistência a pacientes intubados e de emissão de gotículas a partir daqueles sem intubação, o que deve seguramente contribuir para a redução dos riscos de transmissão do vírus para a equipe de profissionais de saúde em UTI^(^^5^^)^ (Tabela 1).

**Tabela 1:** Colutórios utilizados no tratamento de pacientes com COVID-19 internados em unidade de terapia intensiva

Colutórios recomendados	Concentração	Ações principais
C31G (SAVVY^®^)		Redução de microrganismos bucais em pacientes com COVID-19
Cloreto de cetilpiridínio	0,05%	Possível efeito antiviral sobre o SARS-CoV-2
Iodopovidona	0,2%,0,4%, ou 0,5%	Redução da carga viral do SARS-CoV-2, contribuindo para o controle da higiene bucal e do trato respiratório
Peróxido de hidrogênio	1,5% ou 3%	Redução da carga viral salivar do SARS-CoV-2
Clorexidina	0,12%	Supressão do SARS-CoV-2 por 2 horas após o uso, melhora dos sintomas em pacientes com COVID-19 apresentando alteração da flora bucal, e redução de pneumonia associada à ventilação mecânica

Fonte: adaptado de Moosavi MS, Aminishakib P, Ansari M. Antiviral mouthwashes: possible benefit for COVID-19 with evidence-based approach. J Oral Microbiol. 2020; 12(1):1794363. Review;^(^^5^^)^ Vergara-Buenaventura A, Castro-Ruiz C. Use of mouthwashes against COVID-19 in dentistry. Br J Oral Maxillofac Surg. 2020;58(8):924-7. Review.^(^^6^^)^

Outro ponto importante diz respeito ao uso de colutórios nesse grupo de pacientes no intuito de contribuir para a melhora de problemas sistêmicos associados à flora microbiana bucal, assim como para a prevenção da ocorrência de pneumonia nosocomial associada à ventilação mecânica, levando à provável redução do tempo de internação^(^^5^^)^ (Figura 1). É esperado também o benefício relacionado à possibilidade de redução do risco de infecção cruzada entre pacientes internados durante a pandemia.^(^^6^^)^

**Figura 1:** Recomendações para técnica de higienização bucal em pacientes com COVID-19 admitidos em unidade de terapia intensiva. Setas vermelhas: aplicáveis para pacientes dentados ou parcialmente dentados; setas verdes: aplicáveis para pacientes edêntulos; setas pretas: aplicáveis para ambos os grupos

Fonte: adaptado de Agência Nacional de Vigilância Sanitária (ANVISA). Gerência de Vigilância e Monitoramento em Serviços de Saúde. Gerência Geral de Tecnologia em Serviços de Saúde. Medidas de prevenção de infecção relacionada à assistência à saúde. v. 4. Brasília (DF): Ministério da Saúde; 2017. p. 92 [Série Segurança do Paciente e Qualidade em Serviços de Saúde] [citado 2020 Dez 26]. Disponível em: https://www20.anvisa.gov.br/segurancadopaciente/index.php/publicacoes/item/caderno-5;^(^^7^^)^ Conselho Federal de Odontologia (CFO). Recomendações AMIB/CFO para atendimento odontológico COVID-19: Comitê de Odontologia AMIB/CFO de enfrentamento ao COVID-19. Brasília (DF): AMIB/CFO; 2020 [citado 2020 Dez 26]. Disponível em: http://www.crosp.org.br/uploads/arquivo/d45f45bec26af5e60711423292623321.pdf.^(^^8^^)^ UTI: unidades de terapia intensiva.

Vale ressaltar que, para a realização de higienização bucal efetiva em pacientes com COVID-19 admitidos em UTI, alguns critérios devem ser cuidadosamente seguidos. É de suma importância que cada instituição de saúde se adeque dentro da sua realidade quanto à combinação de colutórios escolhidos. Na figura 1, descrevemos uma proposta de passos para a realização desse procedimento.

Destaca-se, além disso, dentro desta proposta, que a recomendação de posicionamento dos pacientes em decúbito elevado visa sobretudo evitar casos de pneumonia decorrente de infecções relacionadas à assistência à saúde, bem como prover melhoria dos parâmetros respiratórios.^(^^7^^)^ Além disso, após a aspiração inicial de secreções acima do *cuff* nos pacientes intubados, recomenda-se o uso de tampão de gaze para complementar a proteção pulmonar, que deve ser realizada antes do uso de colutórios.^(^^8^^)^

Entretanto, mesmo com importantes avanços nos protocolos para o manejo de pacientes com COVID-19 internados em UTI, as equipes profissionais envolvidas devem sempre priorizar a realização de procedimentos com menor risco de gerar aerossóis ou emissão de gotículas, o que corrobora uma maior proteção no ambiente hospitalar. Ainda, a atuação de dentistas intensivistas torna-se essencial à assistência integral a esses pacientes.
